# Trends in Cancer Diagnoses Among People Living with HIV: A 20-Year Retrospective Study from a Tertiary Center in Thailand

**DOI:** 10.3390/jcm15010022

**Published:** 2025-12-19

**Authors:** Jirapat Wonglhow, Supakorn Chaiwiriyawong, Patrapim Sunpaweravong, Chirawadee Sathitruangsak, Arunee Dechaphunkul

**Affiliations:** Division of Medical Oncology, Department of Internal Medicine, Faculty of Medicine, Prince of Songkla University, Songkhla 90110, Thailand; jirapat.jw@gmail.com (J.W.); james.deli.chai@gmail.com (S.C.); spatrapi@medicine.psu.ac.th (P.S.); sjirawadee@gmail.com (C.S.)

**Keywords:** human immunodeficiency virus (HIV), malignancy, AIDS-defining cancer, non-AIDS-defining cancer, prevalence, antiretroviral therapy, trend, people living with HIV (PLWH)

## Abstract

**Background**: Cancer epidemiology data for people living with human immunodeficiency virus (PLWH) in Thailand, particularly in the era of combination antiretroviral therapy (ART), remain limited. In this study, we describe the prevalence, temporal trends, clinical characteristics, and survival outcomes of patients with AIDS-defining cancers (ADCs) and non-AIDS-defining cancers (NADCs). **Methods**: We retrospectively reviewed adult PLWH diagnosed with malignancy at Songklanagarind Hospital in Thailand during 2003–2023. Demographic, human immunodeficiency virus (HIV)-related, and clinical data were analyzed using chi-square and Wilcoxon rank-sum tests and the Kaplan–Meier method. **Results**: Among 444 patients, 231 had NADCs and 213 had ADCs. The NADC proportion increased markedly over time. Common ADCs included non-Hodgkin lymphoma and cervical cancer; common NADCs included lung cancer, non-nasopharyngeal head and neck cancer, and hepatocellular carcinoma. Compared with patients with ADCs, those with NADCs were older, more often male, and had higher proportions of undetectable HIV viral load, CD4 counts ≥200 cells/µL, and ART use. Approximately one-third of patients presented with advanced-stage disease, and the median overall survival was 15.9 months. **Conclusions**: Over two decades, NADCs have become the predominant malignancy in Thai PLWH, associated with older age, male sex, and improved immune function. This reflects the evolving cancer risk in the era of combination ART. We suggest employing multidisciplinary approaches involving HIV and cancer care to improve survival outcomes and integrating age-appropriate screening for common NADCs into HIV care.

## 1. Introduction

Human immunodeficiency virus (HIV) remains a major global health concern, with 40 million people living with HIV (PLWH) as of 2021, up from 29.5 million in 2010 [1]. Although expanded access to combination antiretroviral therapy (ART) has reduced acquired immunodeficiency syndrome (AIDS)-related mortality worldwide [2,3], PLWH continue to experience higher mortality than the general population [4,5,6]. In Thailand, approximately 90% of PLWH are receiving combination ART [7]. As survival improves, cancer has emerged as a growing cause of morbidity and mortality in this population [8,9].

In the era of combination ART, the incidence of AIDS-defining cancers (ADCs), including Kaposi sarcoma, non-Hodgkin lymphoma, and cervical cancer, has declined, whereas non-AIDS-defining cancers (NADCs), such as lung, liver, and anal cancers, have become more prevalent [10,11,12,13,14]. This shift is thought to reflect chronic immune activation, persistent inflammation, and co-infection with oncogenic viruses. The association between immune suppression and ADC risk is well established, whereas the role of immune suppression in NADCs remains uncertain [15,16,17].

Cancers in PLWH often present at a younger age, with atypical pathology, aggressive clinical behavior, and advanced disease stage, all contributing to poorer outcomes [12,18]. Delayed diagnosis, comorbidities, and immunosuppression further increase mortality risk [19]. Guidelines recommend that PLWH receive the same cancer therapies as patients without HIV, with careful attention to drug interactions, supportive care, and treatment-related toxicities [20,21]. However, in real-world settings, disparities persist in access to systemic cancer treatment. PLWH remain an understudied population in clinical trials, with limited evidence to guide optimal cancer management. Routine cancer screening is also less common in PLWH than in the general population, and HIV-specific screening guidelines are lacking [12,22].

In Thailand, cancer has become a leading cause of death in the general population, and the burden of malignancy among PLWH is an increasing concern [23,24]. HIV care in Thailand has expanded substantially with nationwide ART coverage [7], yet important challenges remain. Structural and financial barriers, together with persistent HIV-related stigma, may delay both HIV care and cancer care, which can contribute to an advanced stage at presentation and poorer outcomes in PLWH. Cancer epidemiology data for PLWH is generally sparse in Asia [23,24,25] and extremely limited in Southeast Asia. This paucity of regional data underscores the need for robust datasets to better understand cancer trends in these areas.

Therefore, we conducted a 20-year retrospective study of PLWH with cancer at a tertiary referral center in southern Thailand. The aim of this study was to determine temporal trends in cancer diagnoses, compare the clinical characteristics of ADCs and NADCs, and evaluate survival outcomes in this cohort. By improving our understanding of long-term trends in both ADCs and NADCs, this study can help guide prevention, screening, and management strategies.

## 2. Materials and Methods

### 2.1. Study Design and Participants

This retrospective study was conducted at Songklanagarind Hospital between January 2003 and June 2023 by reviewing electronic medical records. Eligible patients were (1) aged 18 years or older, (2) diagnosed with HIV infection, and (3) diagnosed with any malignancy. Patients whose cancer diagnosis preceded their HIV diagnosis by more than three months were excluded.

We preliminarily identified eligible patients from the hospital information system using ICD-10 codes B20–B24 for HIV infection and C00–C97 for malignant neoplasms. We then confirmed HIV infection and cancer diagnoses by reviewing medical records and pathology reports. All consecutive patients who met the eligibility criteria during the study period were included in the analysis.

### 2.2. Data Collection

Baseline clinical characteristics included age, sex, comorbidities, body mass index, and laboratory results such as CD4 cell count and HIV viral load. Additional HIV-related information, including ART, opportunistic infections, and hepatitis co-infections, was also collected. Antiretroviral therapy status at cancer diagnosis was classified according to whether the patient had received ART before the time of cancer diagnosis, irrespective of the specific regimen or duration. Cancer-specific data, including histological subtype and stage, were also retrieved. All data were obtained from the hospital information system at Songklanagarind Hospital.

AIDS-defining cancers were classified as Kaposi sarcoma, non-Hodgkin lymphoma, or cervical cancer, in accordance with the Centers for Disease Control and Prevention definition. All other malignancies were categorized as NADCs. Staging was determined according to the relevant classification system for each cancer type at the time of diagnosis. Accordingly, TNM-based systems were used for most solid tumors, Barcelona Clinic Liver Cancer staging was used for hepatocellular carcinoma, the Ann Arbor classification was used for lymphomas, and FIGO staging was used for cervical cancer.

### 2.3. Measurement

The primary outcome was the distribution and temporal trends of ADCs and NADCs among PLWH in this hospital-based cohort. Secondary outcomes included identification of the clinical characteristics of ADCs and NADCs, cancer stage at presentation, and survival outcomes. Overall survival (OS) was defined as the time from cancer diagnosis to death from any cause. Patients who remained alive at the last follow-up were censored at that date.

### 2.4. Statistical Analysis

Continuous variables were presented as medians with interquartile ranges (IQRs) or means with standard deviations, depending on the distribution. Categorical variables were summarized as frequencies and percentages. Comparisons between ADC and NADC groups were performed using the chi-square test or Fisher’s exact test for categorical variables and the Wilcoxon rank-sum test for continuous variables. Survival estimates were generated using the Kaplan–Meier method and compared using the log-rank test. For selected comparisons, univariable Cox proportional hazards regression models were used to obtain hazard ratios (HRs) with 95% confidence intervals (CIs) corresponding to the log-rank comparisons. All analyses were performed using R software version 4.5.0 (R Foundation for Statistical Computing, Vienna, Austria). A two-sided *p*-value < 0.05 was considered statistically significant.

### 2.5. Ethical Approval

This study was approved by the Ethics Committee of the Research Centre, Faculty of Medicine, Prince of Songkla University (REC.66388141). Owing to the retrospective design, the requirement for written informed consent was waived. All patient identifiers were removed to ensure confidentiality and protect patient privacy.

## 3. Results

### 3.1. Patient Characteristics

From 2003 to 2023, a total of 444 patients with HIV and cancer were included in this study, comprising 231 (52.0%) with NADCs and 213 (48.0%) with ADCs. Their baseline characteristics are summarized in Table 1.

Patients with NADCs were significantly older than those with ADCs (median age, 47 years [IQR, 41.1–54.7] vs. 39.1 years [IQR 33.3–47.6]; *p* < 0.001). Male sex was dominant in the NADC group (73.6%), whereas female sex dominated in the ADC group (52.6%; *p* < 0.001).

The immunological status also differed between groups. More patients with ADCs than with NADCs had baseline CD4 counts <200 cells/µL (57.3% vs. 34.0%; *p* < 0.001). Similarly, detectable HIV viral load was more frequent in patients with ADCs (22.5% vs. 15.2%; *p* < 0.001).

Regarding comorbidities, cirrhosis was significantly more common in patients with NADCs than in those with ADCs (16.9% vs. 7.0%; *p* = 0.003). Hepatitis C virus co-infection was also more frequent in the NADC group (18.2% vs. 10.3%; *p* = 0.002). The prevalence of other comorbidities, including diabetes, hypertension, dyslipidemia, chronic obstructive pulmonary disease, cardiovascular disease, cerebrovascular disease, hepatitis B virus co-infection, and syphilis, did not differ significantly between groups.

Most patients received ART, although the proportion was higher in the NADC group than in the ADC group (87.4% vs. 74.6%; *p* < 0.001). Opportunistic infections were observed in approximately one-quarter of patients in both groups (26.0% vs. 25.8%; *p* = 1.0). Further details regarding HIV viral load and opportunistic infections are presented in Supplementary Table S1.

### 3.2. Time from HIV Diagnosis to Cancer Diagnosis

Exactly 307 of 444 patients (69.1%) were diagnosed with cancer after HIV diagnosis; a total of 174 had NADCs (56.7%) and 133 had ADCs (43.3%). Further details regarding the proportion of patients diagnosed with cancer associated with the time of HIV diagnosis are presented in Supplementary Table S2. The median time from HIV diagnosis to cancer diagnosis was 60.0 months (range, 0.07–306.3 months). Patients with ADCs developed cancer significantly earlier after HIV diagnosis than those with NADCs (median, 34.1 vs. 84.7 months, *p* < 0.001).

### 3.3. Cancer Distribution and Temporal Trends

**Figure 1:**
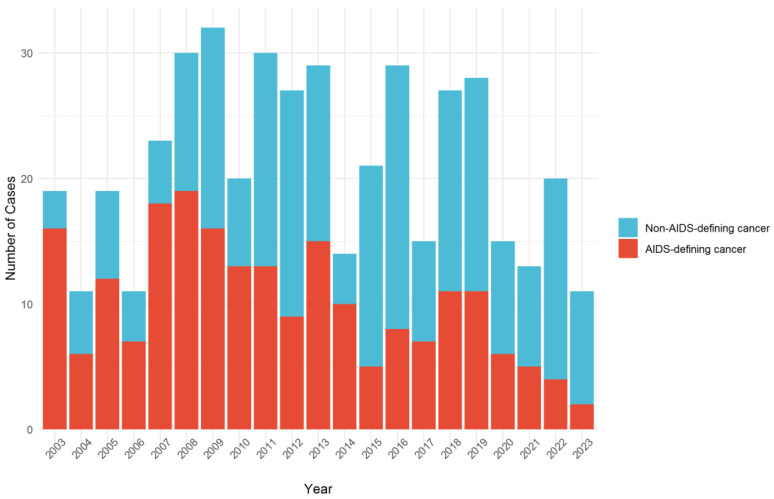
Yearly distribution of non-AIDS-defining and AIDS-defining cancer cases during the study period.

**Figure 2:**
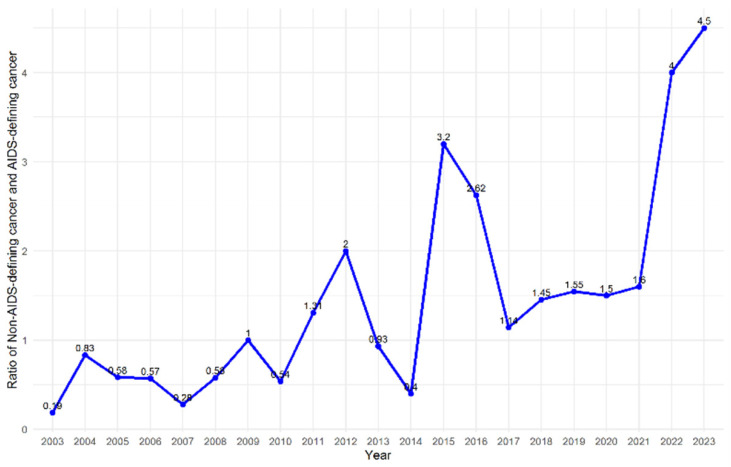
Yearly ratio of non-AIDS-defining to AIDS-defining cancer cases during the study period.

The annual case distribution from 2003 to 2023 showed a gradual increase in the number of NADC cases relative to ADC cases over time (Figure 1). In the early years of the study period (2003–2007), ADCs accounted for the majority of cancer diagnoses. However, from 2010 onward, the proportion of NADC cases gradually increased, and after 2014, NADC cases consistently outnumbered ADC cases each year. In the final study period (2019–2023), NADCs were the predominant cancer type, with a widening gap compared to ADCs. The yearly ratio of NADC cases to ADC cases remained below 1.0 until 2009, fluctuated around 1.0–2.0 between 2010 and 2013, and exceeded 2.0 from 2014 onward, with the highest ratio observed in 2023 (4.5) (Figure 2).

**Figure 3:**
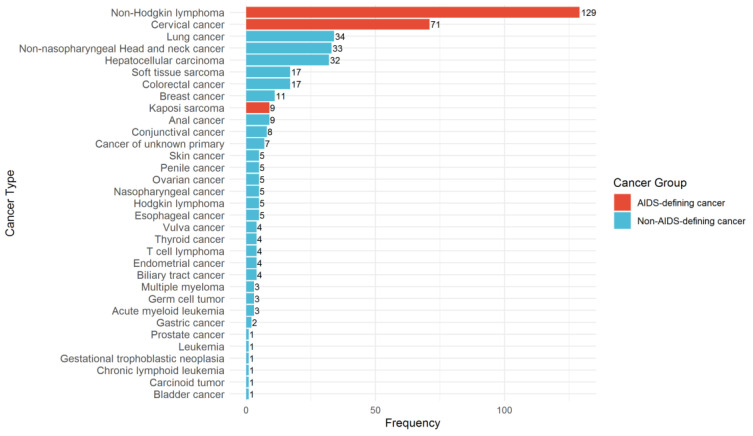
Prevalence of each cancer type in patients living with HIV from 2003 to 2023.

Across the entire cohort, non-Hodgkin lymphoma was the most common cancer (*n* = 129, 29.1%), followed by cervical cancer (*n* = 71, 16.0%) (Figure 3). The most frequent types of NADCs were lung cancer (*n* = 34, 7.7%), non-nasopharyngeal head and neck cancers (*n* = 33, 7.4%), and hepatocellular carcinoma (*n* = 32, 7.2%). Other cancer types included colorectal cancer (*n* = 17, 3.8%), soft tissue sarcoma (*n* = 17, 3.8%), and breast cancer (*n* = 11, 2.5%), along with a wide spectrum of less frequent malignancies. At presentation, 33.1% of the entire cohort were diagnosed with advanced-stage disease. Advanced-stage disease was more frequent among patients with NADCs than those with ADCs (35.5% vs. 30.5%; *p* = 0.311). Information on cancer stage at diagnosis was available for 435 patients (97.97%); nine patients (2.03%) had unknown or undocumented stages.

### 3.4. Survival Outcomes

#### 3.4.1. Overall Survival

In the entire cohort of 444 patients, the median follow-up time was 11.58 months (IQR; 3.74, 37.89). The median OS was 15.9 months (95% CI, 12.0–20.1). The 1-, 3-, and 5-year survival rates were 55.0% (95% CI, 50.3–60.0), 35.8% (95% CI, 31.4–41.2), and 28.6% (95% CI, 24.5–34.1), respectively. Of the 444 patients, 79 (17.8%) did not receive any cancer treatment, whereas the remainder underwent cancer therapy—including surgery, radiotherapy, chemotherapy, or combined modalities—depending on their cancer type and stage (Supplementary Table S3).

In terms of survival outcomes, when stratified by CD4 level, patients with CD4 levels ≥ 200 cells/µL had a median OS of 22.47 months (95% CI, 15.9–34.4), compared with 14.06 months (95% CI, 10.28–19.7) for those with CD4 levels < 200 cells/µL. However, this difference was not statistically significant (HR, 0.86; 95% CI, 0.67–1.11; *p* = 0.243; Supplementary Figure S1). When stratified by HIV viral load, patients with detectable levels had a median OS of 16.9 months (95% CI, 10.6–27.1), compared with 28.6 months (95% CI, 16.5–43.8) for those with undetectable levels (HR, 0.70; 95% CI, 0.51–0.98; *p* = 0.0367; Supplementary Figure S2).

#### 3.4.2. Overall Survival by Cancer Group

**Figure 4:**
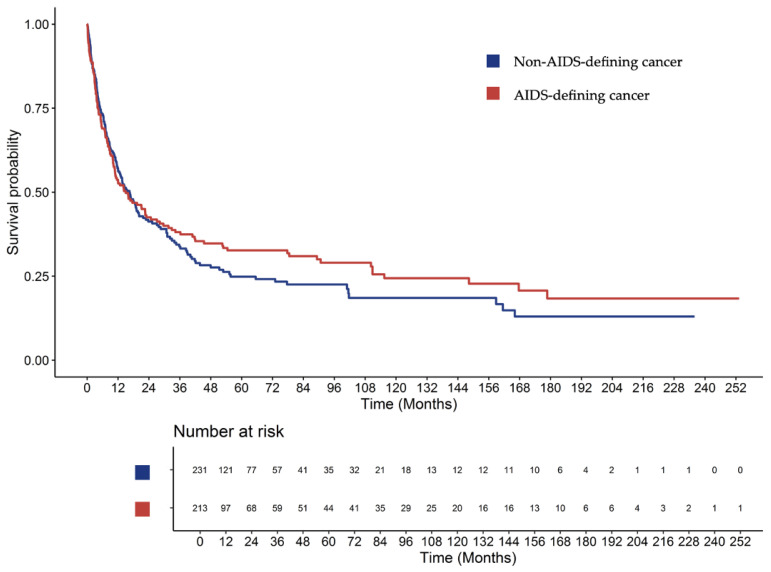
Overall survival between patients with non-AIDS-defining and AIDS-defining cancers.

When stratified by cancer group, patients with ADCs had a median OS of 14.9 months (95% CI, 10.7–24.7), compared with 16.5 months (95% CI, 12.0–21.7) for those with NADCs. However, this difference was not statistically significant (HR, 0.91; 95% CI, 0.73–1.15; *p* = 0.428) (Figure 4). The 1-, 3-, and 5-year survival rates were 53.1% (95% CI, 46.5–60.8), 38.1% (95% CI, 31.2–45.7), and 32.7% (95% CI, 26.5–40.9) for ADCs, and 56.6% (95% CI, 50.3–63.5), 33.8% (95% CI, 27.8–41.1), and 24.9% (95% CI, 18.9–31.8) for NADCs, respectively.

Subtype-specific analyses were performed for the five most frequent cancers with ≥30 cases. These analyses were descriptive rather than comparative owing to the small number of patients in each subgroup, particularly after stage stratification. Thus, the HRs and p-values were not calculated.

For non-Hodgkin lymphoma (*n* = 129), the median OS was 8.08 months (95% CI, 5.45–12.7), with stage-specific survival of 32.88, 12.71, 11.17, and 5.58 months for stages I, II, III, and IV, respectively (Supplementary Figure S3). Cervical cancer (*n* = 71) was associated with a median OS of 36.2 months (95% CI, 22.5–148.0), which decreased with advancing stage: 148.29, 40.90, 22.54, and 3.65 months for stages I–IV, respectively (Supplementary Figure S4). The median OS for lung cancer (*n* = 34) was 8.8 months (95% CI, 4.24–13.60), whereas that for non-nasopharyngeal head and neck cancer (*n* = 33) was 18.6 months (95% CI, 13.6–NA). The median OS for hepatocellular carcinoma (*n* = 32), which was associated with the poorest survival among these subtypes, was 3.61 months (95% CI, 1.81–8.74).

## 4. Discussion

This 20-year retrospective cohort provides a comprehensive overview of cancer among PLWH in southern Thailand. We observed a clear temporal shift in the cancer spectrum, with a decline in ADCs and an increasing predominance of NADCs in the combination ART era. AIDS-defining cancers tended to occur earlier after HIV diagnosis, whereas NADCs developed later, reflecting aging and prolonged survival. Most patients presented at advanced stages and the OS remained poor, with hepatocellular carcinoma showing particularly unfavorable outcomes.

Our findings align with reports from the United States and Europe, which have consistently shown marked reductions in ADC cases, particularly Kaposi sarcoma and non-Hodgkin lymphoma, alongside a rising burden of NADC cases such as lung, liver, and head and neck cancers [26,27,28,29,30]. In Thailand, a 2004 study reported ADC predominance [31], whereas a 2023 study demonstrated NADC predominance [32]. Our study strengthens this evidence by illustrating a long-term, sustained shift from ADC to NADC predominance. In contrast, findings from pediatric cohorts indicate that ADCs remain the most common malignancy [33,34]. This suggests that the temporal shift in adults is likely driven by combination ART-mediated survival gains, which allow age- and lifestyle-related cancers to emerge [16]. The clinical characteristics of our cohort further support this interpretation. Patients with NADCs were older, had higher CD4 counts, and were more likely to have virologic suppression and ART exposure.

The shift from ADCs to NADCs has important clinical and public health implications. As PLWH live longer, cancer prevention and screening strategies must adapt to this changing malignancy spectrum. At present, no cancer screening guidelines are specifically tailored to PLWH. Beyond aging and lifestyle factors, HIV infection itself may be a risk factor for cancer, particularly infection-related malignancies, which remain more frequent in PLWH than in individuals without HIV [16,35,36,37]. Comorbidities and co-infections—such as cirrhosis and hepatitis C, which were more common among patients with NADCs in our study—likely contributed further to cancer risk, consistent with the strong association between viral hepatitis and hepatocellular carcinoma [38]. Cervical, lung, and liver cancers were the most common solid tumors in our cohort, with one-third of patients presenting at advanced stages and experiencing poor survival outcomes. The high proportion of advanced-stage cancers in our cohort is likely multifactorial. Many patients are diagnosed with HIV only when they present with symptomatic illness, leaving little opportunity for early cancer detection. Routine cancer screening for PLWH is not systematically integrated into HIV clinics, especially for NADCs, and awareness of cancer risk among patients and providers may be limited. Furthermore, stigma, socioeconomic constraints, and limited availability of imaging or endoscopic investigations in some settings may delay presentation and diagnostic work-up, contributing to a late stage at diagnosis and poorer survival. These findings highlight the importance of early detection and prevention strategies. Specifically, cancer screening, human papillomavirus vaccination, and healthy lifestyle behaviors, including eating a balanced diet, performing regular physical activity, avoiding heavy alcohol consumption, and stopping smoking, should be encouraged, as in the general population [11,39,40,41,42,43]. Whether PLWH would benefit from earlier or more intensive screening than individuals without HIV remains uncertain and warrants future research.

In Thailand, several health-system factors may have contributed to the observed cancer patterns among PLWH. Access to organized cancer screening remains limited, particularly for NADCs, and specialist oncology services are concentrated in large urban tertiary centers. Decentralization of ART services to peripheral hospitals, although beneficial for HIV control, may fragment care pathways between HIV clinics and oncology units and result in fewer cancer referrals to tertiary centers. In addition, structural and financial barriers, including travel distance, transportation costs, and loss of income, together with persistent HIV- and cancer-related stigma, may delay both HIV and cancer diagnosis and reduce the likelihood of a timely oncologic treatment onset.

In our cohort, patients with virologic suppression appeared to have better OS in unadjusted analyses, supporting the importance of achieving and maintaining virologic suppression. However, this exploratory survival finding should be interpreted cautiously given the heterogeneity of cancer types, stages, and treatments. A previous study revealed disparities in cancer treatment between PLWH and patients without HIV, which may contribute to lower survival rates [44]. Contemporary evidence supports the use of standard oncology treatments—including surgery, radiotherapy, chemotherapy, targeted agents, and immune checkpoint inhibitors—in PLWH when drug–drug interactions and toxicities are properly managed [10,12,20,45]. Although our study lacked a direct HIV-negative comparator, our findings are hypothesis-generating and suggest that immune reconstitution under combination ART may facilitate both access to and tolerance of curative-intent therapies, thereby improving clinical outcomes. Collectively, well-controlled PLWH may achieve clinical outcomes comparable to those of patients without HIV when treated with guideline-concordant therapy. Multidisciplinary approaches that involve HIV and cancer care are required [46]. More importantly, HIV serostatus alone should not justify dose reductions, treatment delays, or exclusion from clinical trials [45,47]. Trial eligibility should be based on clinical stability, such as sustained viral suppression and adequate CD4 counts [48]. A broader inclusion of PLWH in oncology trials may generate valuable evidence specific to this growing population.

Our study has several strengths, including our adoption of one of the largest single-center cohorts of cancer among PLWH in Southeast Asia, the 20-year observation period, and the inclusion of detailed clinical and immunologic data. Nonetheless, certain limitations must be acknowledged. The retrospective, single-center design may limit generalizability, and missing data for some variables could introduce bias. The apparent decline in cancer cases in recent years may not necessarily reflect a true decrease in incidence but could be partly due to health policy changes that decentralized ART dispensing and HIV care to local hospitals, reducing referrals to our center. A similar decentralization may have occurred in cancer management, with more patients being treated closer to home. Therefore, these findings should be interpreted with caution, as they reflect a hospital-based cohort and may not represent population-based incidence trends. In particular, comprehensive longitudinal data on the size of the underlying HIV clinic population or person-time at risk were not available; therefore, we were unable to distinguish true changes in cancer incidence from shifts in referral patterns or survival duration. In addition, because our study spans 20 years, HIV testing strategies, ART regimens, and cancer diagnostic modalities likely changed substantially over this period. These secular changes may have influenced both the prevalence and stage distribution of cancers, as well as survival outcomes among PLWH. However, detailed information on testing policies, specific ART regimens, and diagnostic technologies was not systematically collected in our dataset; therefore, we could not formally adjust for or analyze these effects. Furthermore, cancer heterogeneity by type and stage, as well as incomplete treatment information, limit the interpretation of survival analyses. Finally, the absence of an HIV-negative comparator precludes direct estimation of the HIV-attributable cancer risk.

Future prospective studies should include patients with and without HIV to clarify the independent contribution of HIV to cancer risk and optimize screening strategies for this high-risk population. Additionally, further research in specific cancer subtype cohorts will help better define treatment outcomes and survival differences and inform evidence-based policies promoting equitable access to cancer care and appropriate inclusion of PLWH in oncology clinical trials.

## 5. Conclusions

This 20-year retrospective cohort demonstrates a clear shift in the cancer spectrum among PLWH in Thailand, with NADC cases increasingly surpassing ADC cases in the era of combination ART. Most patients presented with advanced-stage disease, and survival remained poor, although outcomes varied across cancer subtypes. Clinical characteristics—including age, sex, immune status, and comorbidities—were distinct between ADC and NADC cases, underscoring divergent risk profiles. Our findings highlight the importance of integrating cancer prevention and age-appropriate screening for common NADCs into HIV care. These results are hypothesis-generating and align with prior evidence suggesting that PLWH with an undetectable viral load and good immune recovery can benefit from standard oncologic therapies. Multidisciplinary approaches involving HIV and cancer care are crucial for improving the survival outcomes of PLWH with cancer.

## Figures and Tables

**Table 1:** Baseline characteristics of patients with NADCs and ADCs.

Characteristic	NADCs (*n* = 231)	ADCs (*n* = 213)	Total (*n* = 444)	*p*-Value
Sex, *n* (%)				<0.001
Male	170 (73.6)	101 (47.4)	271 (61.0)	
Female	61 (26.4)	112 (52.6)	173 (39.0)	
Age				
Median, years (IQR)	47 (41.1, 54.7)	39.1 (33.3, 47.6)	43.1 (36.7, 52.3)	<0.001
≥60 years, *n* (%)	26 (11.3)	13 (6.1)	39 (8.8)	0.08
BMI, *n* (%)				0.312
<18.5 kg/m^2^	74 (32.0)	67 (31.5)	118 (26.6)	
18.5–22.99 kg/m^2^	98 (42.4)	83 (39.0)	150 (33.8)	
≥23.0 kg/m^2^	57 (24.7)	56 (26.3)	103 (23.2)	
NA	2 (0.9)	7 (3.3)	9 (2.0)	
CD4 level, *n* (%)				<0.001
<200 cells/μL	65 (28.1)	98 (46.0)	163 (36.7)	
≥200 cells/μL	126 (54.6)	73 (34.3)	199 (44.8)	
NA	40 (17.3)	42 (19.7)	82 (18.5)	
Viral load, *n* (%)				<0.001
Detectable	35 (15.2)	48 (22.5)	83 (18.7)	
Undetectable	103 (44.6)	47 (22.1)	150 (33.8)	
NA	93 (40.2)	118 (55.4)	211 (47.5)	
Underlying disease, *n* (%)				
DM	14 (6.1)	19 (8.9)	33 (7.4)	0.334
Hypertension	23 (10.0)	24 (11.3)	47 (10.6)	0.769
Dyslipidemia	28 (12.1)	22 (10.3)	50 (11.3)	0.655
Cirrhosis	39 (16.9)	15 (7.0)	54 (12.2)	0.002
COPD	2 (0.9)	3 (1.4)	5 (1.1)	0.674
Coronary artery disease	1 (0.4)	2 (0.9)	3 (0.7)	0.609
Cerebrovascular disease	2 (0.9)	1 (0.5)	3 (0.7)	1.0
Co-infection, *n* (%)				
HBV	15 (6.5)	17 (8.0)	32 (7.2)	0.673
HCV	42 (18.2)	22 (10.3)	64 (14.4)	0.027
Syphilis	3 (1.3)	1 (0.5)	4 (0.9)	0.625
Opportunistic infection, *n* (%)	60 (26.0)	55 (25.8)	115 (25.9)	1.0
ART, *n* (%)				<0.001
No	27 (11.7)	49 (23.0)	76 (17.1)	
Yes	202 (87.4)	159 (74.6)	361 (81.3)	
NA	2 (0.9)	5 (2.3)	7 (1.6)	

ADCs, AIDS-defining cancers; NADCs, non-AIDS-defining cancers; IQR, interquartile range; DM, diabetes mellitus; HBV, hepatitis B virus; HCV, hepatitis C virus; BMI, body mass index; ART, antiretroviral therapy; COPD, chronic obstructive pulmonary disease; NA, not available.

## Data Availability

The datasets used and/or analyzed in the current study are available from the corresponding author upon reasonable request.

## Supplementary Materials

The following supporting information can be downloaded at https://www.mdpi.com/article/10.3390/jcm15010022/s1. Figure S1: Overall survival between patients with CD4 ≥ 200 cells/μL and CD4 < 200 cells/μL; Figure S2: Overall survival between patients with detectable and undetectable HIV viral loads; Figure S3: Overall survival of patients diagnosed with non-Hodgkin lymphoma stratified by stage; Figure S4: Overall survival of patients diagnosed with cervical cancer stratified by stage; Table S1: Details of HIV viral load and opportunistic infections in patients with NADCs and ADCs; Table S2: Proportion of patients with HIV and cancer diagnosis; Table S3: Cancer treatment modalities among study participants.

## Abbreviations

The following abbreviations are used in this manuscript:

ADC	AIDS-defining cancer
AIDS	acquired immunodeficiency syndrome
ART	antiretroviral therapy
CI	confidence interval
HIV	human immunodeficiency virus
HR	hazard ratio
IQR	interquartile range
NADC	non-AIDS-defining cancer
OS	overall survival
PLWH	people living with HIV
