# Supplementary material for: Trends in Cancer Diagnoses Among People Living with HIV: A 20-Year Retrospective Study from a Tertiary Center in Thailand
Source: J Clin Med. 2025 Dec 19;15(1):22. doi: 10.3390/jcm15010022 (PMC12787150; doi:10.3390/jcm15010022)

Supplementary data

Supplementary Table S1. Details of HIV viral load and opportunistic infections in patients with NADCs and ADCs

	NADCs	ADCs	Total	P-value
Viral load, n (%)	(N=35)	(N=48)	(N=83)	0.072
≤100	5 (14.3)	3 (6.5)	8 (9.9)	
101-1,000	5 (14.3)	10 (21.7)	15 (18.5)	
1,001-10,000	9 (25.7)	4 (8.7)	13 (16.0)	
10,001-100,000	8 (22.9)	8 (17.4)	16 (19.8)	
≥100,001	8 (22.9)	21 (45.7)	29 (35.8)	
Opportunistic infection, n (%)	(N=60)	(N=55)	(N=115)	1.0
PJP	2	4	6	
TB	50	33	83	
Cryptococcosis	0	2	2	
CMV	3	8	11	
Toxoplasmosis	1	1	2	
Histoplasmosis	2	3	5	
Nocardia	1	1	2	
Candidiasis	3	2	5	
Herpes simplex	1	1	2	
Talaromyces	0	1	1	
NTM	1	0	1	

ADCs = AIDS-defining cancers. NADCs = Non-AIDS-defining cancers. PJP = Pneumocystis jirovecii pneumonia. TB = Tuberculosis. CMV = Cytomegalovirus. NTM = Nontuberculous mycobacteria.

Supplementary Table S2. Proportion of patients with HIV and cancer diagnosis

	NADCs	ADCs	Total	P-value
HIV diagnosis before cancer diagnosis	174 (75.3)	133 (62.4)	307 (69.1)	0.012
HIV diagnosis at cancer diagnosis	19 (8.2)	25 (11.7)	44 (9.9)	0.286
HIV diagnosis after cancer diagnosis	38 (16.5)	55 (25.8)	93 (21.0)	0.018

ADCs = AIDS-defining cancers. NADCs = Non-AIDS-defining cancers.

Supplementary Table S3. Cancer treatment modalities among study participants

**Cancer treatment***	**N (%)**
Cancer surgery	86 (19.4)
Radiotherapy	210 (47.3)
Systemic treatment	247 (55.6)
None of the above treatments	79 (17.8)

*could have more than one answer

Supplementary Fig. S1. Overall survival between patients with CD4 ≥200 cells/μL and CD4 <200 cells/μL.

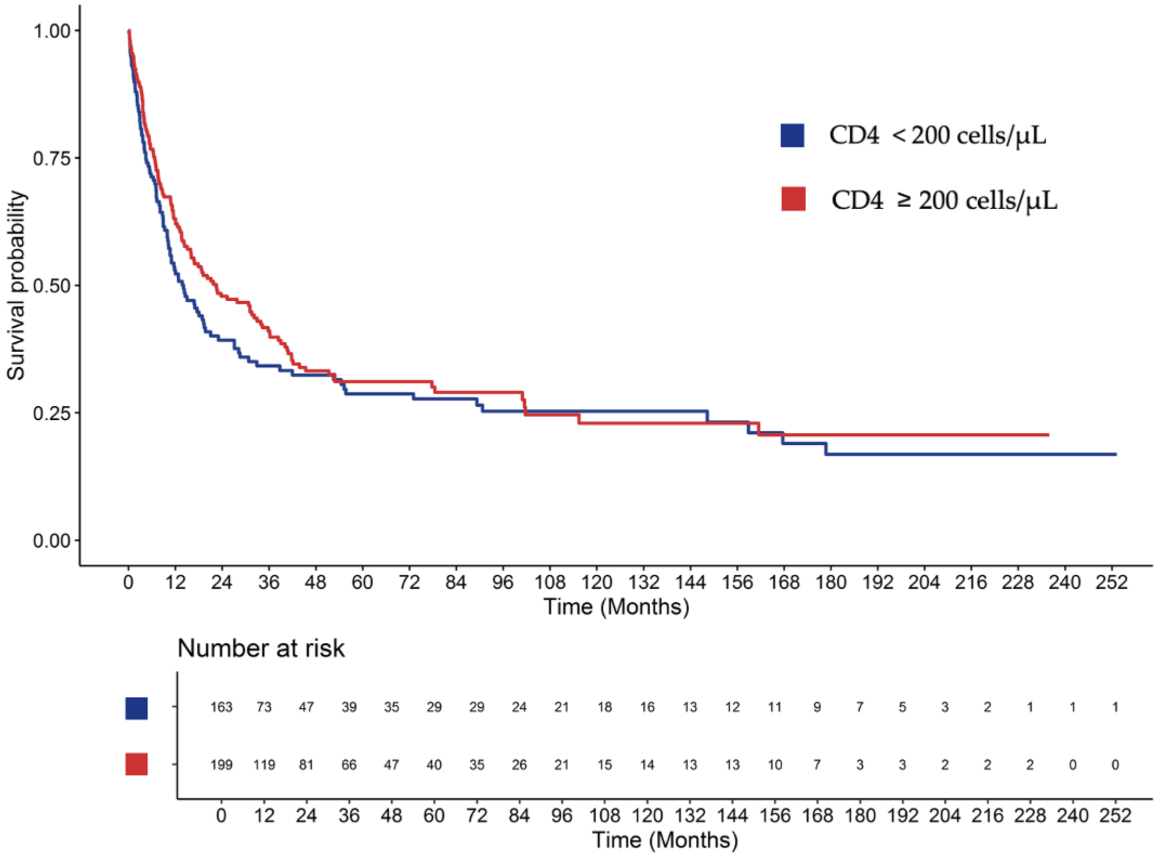

Supplementary Fig. S2. Overall survival between patients with detectable and undetectable HIV viral load.

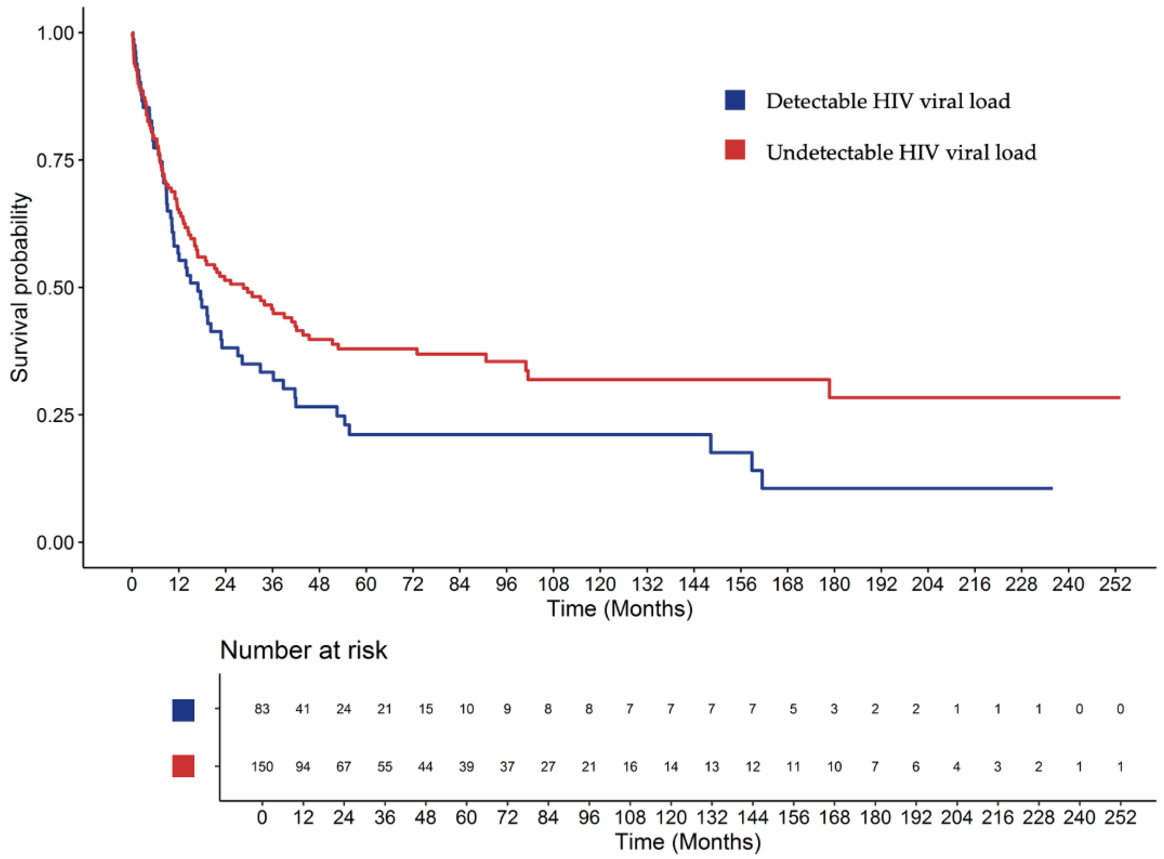

Supplementary Fig. S3. Overall survival of patients diagnosed with non-Hodgkin lymphoma stratified by stage.

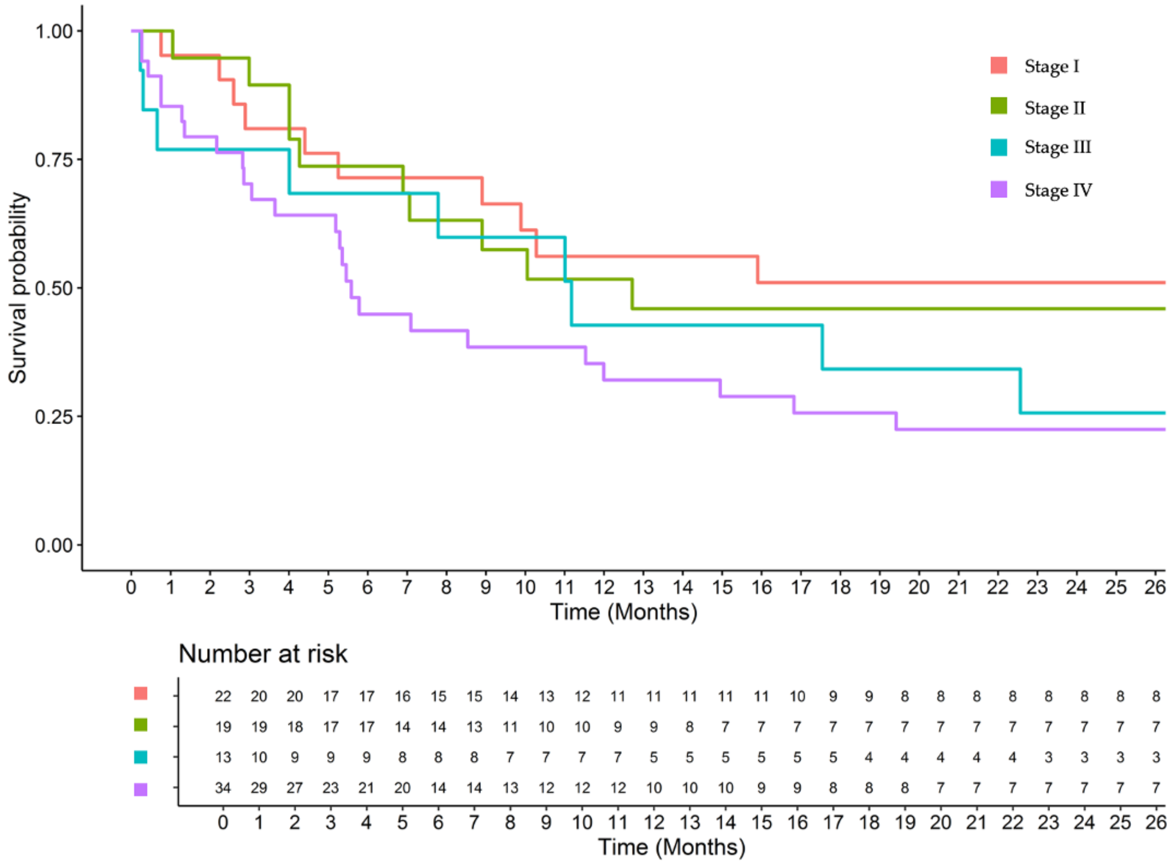

Supplementary Fig. S4. Overall survival of patients diagnosed with cervical cancer stratified by stage.

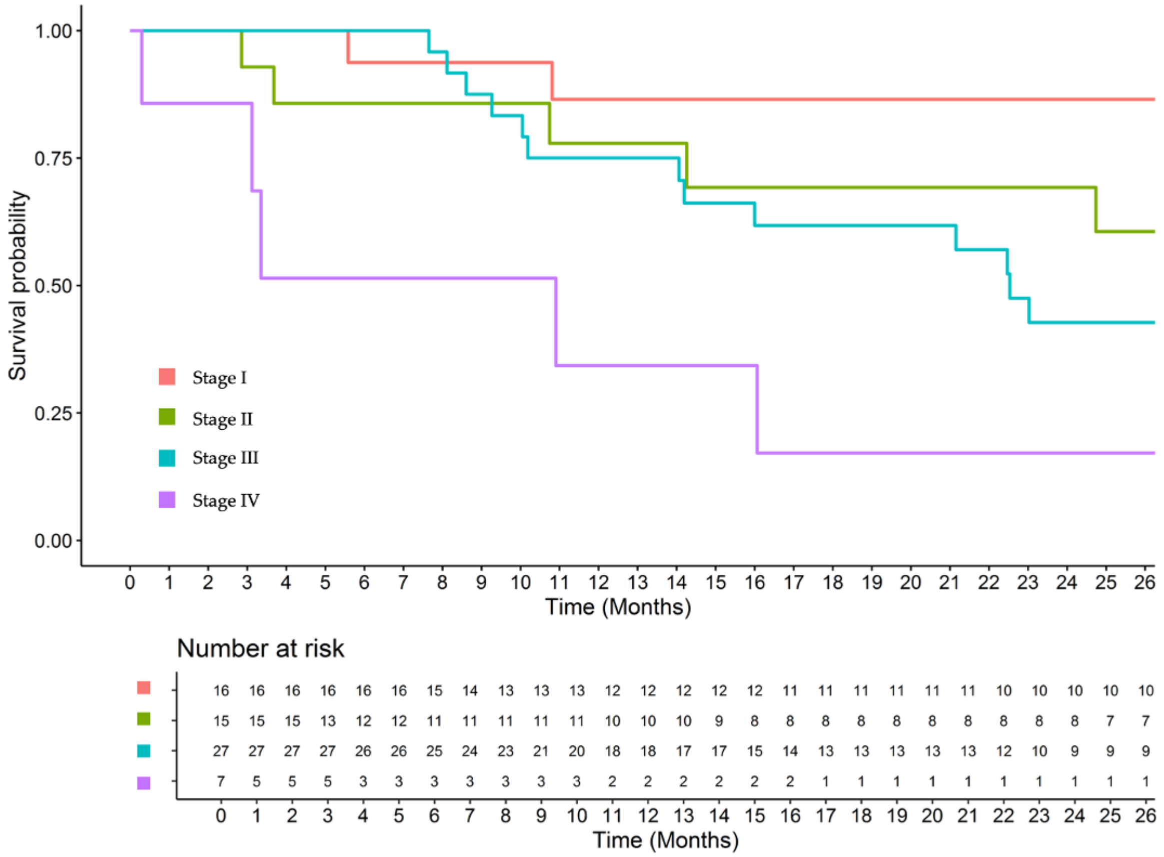

Supplement: Supplementary file 1 [file jcm-15-00022-s001.zip › jcm-3980673-supplementary.pdf]
